# Tuning in to Kids in Schools: a protocol for evaluating an emotion socialization intervention for teachers in Norwegian elementary schools

**DOI:** 10.3389/fpsyg.2026.1618238

**Published:** 2026-03-17

**Authors:** Ada Koleini, Frederik F. Skoe, Christiane E. Kehoe, Thormod Idsoe, Hilde E. Randgaard, Hanne M. Olsen, Ragnhild H. Mjanger, Sophie S. Havighurst

**Affiliations:** 1 Department of Psychology, University of Oslo, Oslo, Norway; 2 District Frogner, Oslo Municipality, Oslo, Norway; 3 Department of Psychiatry, Mindful Centre for Training and Research in Developmental Health, University of Melbourne, Melbourne, VIC, Australia; 4 Department of Special Needs Education, University of Oslo, Oslo, Norway; 5 Pedagogical Psychological Services, Oslo Municipality, Oslo, Norway; 6 Health Agency, Oslo Municipality, Oslo, Norway

**Keywords:** emotion coaching, emotion dismissing, emotion socialization, emotional support, intervention, school, teacher, Tuning in to Kids

## Abstract

Emotional competence allows children to deal with emotional challenges and is a key predictor for mental health and academic outcomes in children. Therefore, improving emotional competence in children is an important endeavor. This protocol outlines how the universal intervention Tuning in to Kids in Schools (TIKiS) will be implemented, and the ways the effectiveness and implementation measures of TIKiS will be evaluated. TIKiS consists of six 1.5 h group sessions aiming to improve the emotional competence and emotion socialization practices of teachers. Using randomized controlled trials, 20 schools in Oslo, Norway participated in a study using TIKiS as either an intervention or waitlist control school. The current study is a collaboration between the University of Oslo, and the municipal agencies that work closely with the schools, to create a sustainable continuation of TIKiS after the study is over. By targeting teachers rather than parents, the competence from TIKiS can remain in the schools and reach more children than a parenting intervention.

**Clinical trial registration:**

https://clinicaltrials.gov, identifier #NCT06501300.

## Introduction

Schools provide the ideal context for offering universal support to children to assist in their development. When children develop emotional competence, skills in identifying, understanding and regulating emotions (Eisenberg et al., 1998; Saarni, 1999) it facilitates their optimal development and helps reduce risks for mental health difficulties (Blewitt et al., 2018; Greenberg et al., 2017), bullying, and aggressive behavior (Espejo-Siles et al., 2020; You et al., 2023). Children’s emotional competence is shaped by emotion socialization experiences, which includes the way parents and teachers role model emotion expression, respond to children’s emotions and teach children about emotions (Eisenberg et al., 1998). Poorer emotion socialization by parents and teachers is associated with more externalizing and internalizing behavior in children (Bjørk et al., 2020; Johnson et al., 2017; Marzano et al., 2003; Trentacosta and Fine, 2010). In the school context, the way teachers interact with children when emotions occur plays an important role in shaping their emotional competence and influences the emotional climate of the classroom. There’s been many examples of socio-emotional learning (SEL) programs aimed at the student population, but none have explicitly focused on emotional skill development in the teachers (Durlak et al., 2011). However, interventions aimed at helping teachers identify, understand and regulate their own emotions, and how they can optimally support children when emotions occur, are less common.

The current paper outlines a protocol for a study testing a universal intervention with teachers in Norwegian primary schools (Tuning in to Kids in Schools: TIKiS). TIKiS aims to support teachers in responding to children’s emotions in ways that improve the emotional climate of the classroom and ultimately improve children’s emotional competence, behavior, social functioning and academic functioning. TIKiS is based on the evidence-based parenting program, Tuning in to Kids® (Havighurst et al., 2010; Havighurst and Harley, 2007), and was adapted and delivered to teachers to help improve their emotion socialization in day-to-day interactions with students.

### Background

Promoting emotional competence in children, especially in their younger years, is a key endeavor of universal interventions because emotional competence has been identified as a key protective factor against the risk for mental health problems (Cicchetti and Hinshaw, 2002). In the past decade there has been an increase in bullying and aggression (Utdanningsforbundet, 2018; Wendelborg, 2023), self-reported symptoms (Bakken, 2022) and diagnosed mental health disorders (Bang et al., 2024; Bråten et al., 2023) in Norwegian children and adolescents. However, the waiting lists for child and adolescent psychiatry are long (Bremnes and Indergård, 2021, 2022), and there is a growing need for universal interventions that may alleviate the pressure on mental health services. Improving emotional competence in schools is one way to address the growing incidence of mental health problems.

In the classroom, teachers are socialization agents who can influence many children and have the potential to be an important conduit to improve child outcomes by promoting emotional competence (Denham et al., 2022). Targeting children’s emotional competence at school may universally promote their ability to recognize, respond to and manage their own emotions and facilitate healthy emotion expression and responses to peer’s emotions. Higher levels of emotion competence in children have been found to be associated with better social and behavioral functioning, academic achievement (Durlak et al., 2011; MacCann et al., 2020; Trentacosta and Izard, 2007), secure attachment (Bender et al., 2015), and mental health outcomes in children, adolescents and young adults (Jones et al., 2015) in studies with various ethnicities in the USA (Durlak et al., 2011; Jones et al., 2015; MacCann et al., 2020; Trentacosta and Izard, 2007), and in Europe (Bender et al., 2015; Denham et al., 2022).

Emotion socialization by parents, teachers and those around a child is the primary way in which environmental influences shape children’s emotional competence. Emotion socialization includes modeling of emotion expression and regulation, responses to children’s emotions, and direct teaching about emotions—such as coaching or guiding children about how to understand and regulate emotions (Eisenberg, 2020; Eisenberg et al., 1998). Emotion socialization is influenced by the socializer’s beliefs and cognitions about emotions (Eisenberg et al., 1998; Gottman et al., 1996) that determine emotional reactions and responses to one’s own and other’s emotions. Their emotional competence and wellbeing also impacts emotion socialization through their ability to engage in healthy emotion expression and respond supportively and calmly to children during emotional moments which also impacts the emotional climate of the child’s home or school environment (Havighurst and Kehoe, 2017; Westrupp and Kehoe, 2024). Moreover, emotion socialization is influenced by culture, for example Norwegian teachers are more likely to use emotion dismissing characterized by distraction rather than punitive reactions (Bjørk et al., 2020; Silkenbeumer et al., 2016).

The most common styles of responding to emotions are emotion coaching and emotion dismissing (Gottman et al., 1996; Katz and Hunter, 2007). Emotion coaching is a supportive response to children’s emotions, characterized by empathy, acceptance, encouragement of children’s emotions and guidance around solving problems and having limits around behaviors. Emotion dismissing on the other hand, is considered unsupportive, and characterized by minimizing, avoidance or punitive reactions to children’s emotions. Supportive emotion socialization is associated with better emotional competence in children; conversely unsupportive emotion socialization is associated with poorer emotional competence, externalizing and internalizing behaviors in kindergarteners and elementary school children in studies from different countries, including Norway (Bjørk et al., 2020; Havighurst et al., 2009; Johnson et al., 2017; Trentacosta and Fine, 2010).

Tuning in to Kids in School (TIKiS) is an intervention that aims to improve teachers’ emotion socialization practices, impacting the emotional climate of the classroom and ultimately supporting the development of children’s emotional competence. TIKiS is based on the Australian evidence-based Tuning in to Kids® parenting program, that has been used to promote parents’ supportive emotion socialization (Havighurst and Harley, 2010). A recent pilot study of TIKiS was conducted with 54 Norwegian teachers and staff from two primary schools. Self-report surveys were completed by 25 intervention and 29 waitlist control teachers/school staff; classroom observations were completed for 13 intervention and 15 control teachers/school staff to assess classroom climate. Results were promising, showing reduced teacher-reported Emotion Dismissing and improved observed CLASS positive emotion climate in the intervention but not the control condition (Bølstad et al., 2023). Moreover, teachers reported that they were satisfied with the intervention and would recommend TIKiS to others (Rivø, 2023). The small sample size prevented detection of significant changes in Emotion Coaching. These results warrant investigation of the effectiveness of this intervention with a larger sample, a longitudinal randomized control design and modifications made to the program after the pilot study.

Implementation is the process of carrying out an intervention; how well an intervention is implemented, influences the effectiveness of the delivery of the content (Durlak and DuPre, 2008; Moir, 2018). For example, if a teacher only participates in half the intended program content, they may not experience the same benefits as a teacher who has engaged with the entire intervention. Moreover, if the teacher rejects the intervention or the implementation is not feasible, it is unlikely to achieve the intended goals. It is, therefore, important to evaluate the implementation of intervention, not just its effectiveness (Durlak and DuPre, 2008; Greenberg et al., 2005; Humphrey et al., 2016).

The current paper outlines the research protocol of a trial to evaluate the effectiveness and implementation quality of TIKiS. The project aimed to:

1. Evaluate the effectiveness of TIKiS, including whether it improves teachers’ own emotion competence and wellbeing, as well as emotion socialization with children and the classroom emotional climate.
2. Evaluate the implementation quality of TIKiS (e.g., program differentiation, reach, fidelity, dosage, adjustment, acceptability, and barriers and enablers of the intervention), and the impact of the intervention on the quality of the relationships within and across the school systems.

## Materials and methods

### Procedure

This study was conducted in Norway, where the elementary school year starts the second or third week of August and ends in the third week of June. Teachers and school leaders use the week before the students start in August to plan the year. A typical Norwegian classroom consists of approximately 20 students, and 1–2 teachers.

In the week before the school year started, baseline questionnaires were completed by leaders and teachers in intervention and control schools. Immediately after filling in the questionnaires, all teachers in grades 1–7 in the intervention schools took part in a three-hour introductory seminar that provided basic information about children’s emotional competence and emotion socialization processes. After this, all teachers in Grade 1–4 of the intervention and control schools were observed using the Classroom Assessment Scoring System K3 (CLASS). The observations and intervention were restricted to grades 1–4 because the K3 version was designed for the 1st to 4th grade classrooms, and due to a limitation in resources for complete school delivery. Teachers in the intervention schools were observed in the first 4 weeks of the school year, to ensure observations were completed before the group delivery of TIKiS. Observations of teachers in the control schools were all conducted within 7 weeks of the start of the new school year. School leadership and teachers were aware of their condition prior to starting evaluation, but those conducting observations of classrooms were blind to the school’s condition.

Once observations were complete, teachers in Grades 1–4 in the intervention condition attended 6 × 1.5-h group TIKiS sessions delivered on a fortnightly basis by trained facilitators from both the school psychological services and the local or school-based health services (2 facilitators for each group). Additionally, each school had three x 90-min TIKiS implementation sessions with school leaders and other staff who had roles of supporting teachers.

Follow-up assessments were administered to intervention and control schools in April/May the following year (5- or 6-month follow-up) and consisted of questionnaires completed by all teachers (Grade 1–7) and CLASS observations of teachers in grades 1–4. Two focus group interviews were conducted in March 2025 with a randomly selected sub-sample of TIKiS facilitators (*n* = 8) and leaders (*n* = 8) from schools in the second year of intervention delivery (3 months after completion of the intervention). See Table 1 for the study overview.

**Table 1:** TIKiS study overview.

Stage of study	Duration	When	Participant
Recruitment		R1: Winter 2022 R2: Winter 2023	Schools
Training of facilitators	2 days × 8 h	R1: Spring 2023 R2: Spring 2024	Facilitators
Allocation to condition		R1: Spring 2023 R2: Spring 2024	Schools
Baseline questionnaires	45 min	R1: August 2023 R2: August 2024	Whole school
Introductory seminar	3 h	R1: August 2023 R2: August 2024	Whole school
Baseline observations	4 × 30 min per teacher	R1: August–October 2023 R2: August–October 2024	Teachers 1–4 Grade
Intervention sessions	6 sessions × 90 min	R1: September–December 2023 R2: September–December 2024	Teachers 1–4 Grade
Implementation support	3 sessions × 90 min	R1: August–October 2023 R2: August–October 2024	School leaders
Supervision for facilitators	3 sessions × 90 min	R1: October–November 2023 R2: October–November 2024	Facilitators
Follow-up questionnaires	45 min	R1: April–June 2024 R2: April–June 2025	Teachers 1–4 Grade
Follow-up observations	4 × 30 min per teacher	R1: April–June 2024 R2: April–June 2025	Teachers 1–4 Grade

R1 = First year of the intervention completed August 2023–June 2024; R1 = Second year of the intervention, completed August 2024 – June 2025.

#### Recruitment and randomization

This study used a randomized controlled trial (RCT) design. Schools (*N* = 20) were matched in pairs using school size and a socioeconomic distribution key (reference?) based on a combination of the median income and level of education of the schools’ parents, and the number of cases reported to child services. The pairs were then randomly allocated to either immediately start intervention or a 1-year wait-list control by an independent third party blinded to school names/details. Schools were randomized to intervention or 1-year waitlist control conditions after all schools for each year were recruited 6-months prior to intervention delivery for the year 2023 (year 1; 10 schools) and 2024 (Year 2; 10 schools).

All schools were recruited from Oslo, which is divided into 15 districts. From the 116 elementary schools in Oslo, 14 schools were excluded because of prior exposure to TIKiS. From a final pool of 112, all schools were invited and 20 schools (18%) agreed to participate in the study. Schools were largely recruited from *Kompetanseløftet for Inkluderende Praksis (The competence lift for inclusive practice)*. Kompetanseløftet is a meeting attended by principals and representatives from schools and kindergartens in Norway to learn of ways to increase competence in these institutions. TIKiS was presented at this meeting because it was consistent with this focus on school competence, and thus fits with an overall strategy of the schools within these districts.

After Kompetanseløftet, interested schools emailed the project team, and were sent a more detailed description about the study and invited to an online information meeting. The deadline for enrolling in the project was 1 week after this online meeting. The final sample of schools were recruited based on the school leaders’ engagement and consent to take part in the intervention.

Recruitment and retention can be difficult in school interventions (other school intervention refs) and were challenging during the pilot study (Bølstad et al., 2023). As such, in the current trial we worked closely at the municipality level and then with the school leaders so that they could support and encourage the teachers to engage in the study. Meetings were held with the school leaders after recruitment and prior to the intervention to plan the next year and to increase engagement. Some schools required extra meetings and support to plan delivery.

### Ethical approval and clinical trial registration

The study received approval from the Ethics Committee of the Psychology Department at the University of Oslo (#26969307) and the Norwegian Agency for Shared Services in Education and Research (SIKT; #734024). School leaders, teachers and group facilitators provided informed consent to participate in the research. The study was also registered with Clinical Trials (#NCT06501300).

### Inclusion/exclusion criteria

Schools were eligible to participate if they had no previous exposure to TIKiS in the pilot study, and they were an elementary school in Oslo. Teachers in Grade 1–4 (children aged 6 to 10 years old) were eligible if they taught structured indoor classes other than gym or swimming, and if they consented to be observed in their classrooms as part of the evaluation. Primarily the teachers were subject teachers, homeroom teachers or resource teachers. Schools were also able to determine whether special education teachers were able to participate.

### TIKiS intervention

TIKiS is an adapted version of the parenting program, *Tuning in to Kids®* (TIK: Havighurst and Harley, 2007), for delivery with elementary school teachers (Havighurst et al., 2023). TIK is a 6-session group program that helps the adult (parent/teacher) develop skills in emotion socialization including understanding and regulating their own emotions and learning emotion coaching for responding to children’s emotions. The five steps of emotion coaching modified for this program are; (1) become aware of the child’s emotions, (2) recognize the child’s emotions as an opportunity for connection, (3) Empathize with and validate the child’s emotions, (4) Help the child label their emotions and (5) Set limits and help the child solve their problems (Gottman et al., 1996). These emotion socialization skills are proposed to help children learn emotional competence, thereby promoting their emotional, social, behavioral and academic functioning and preventing and reducing mental health difficulties (Havighurst et al., 2024).

The original TIK parent manual was translated to Norwegian by Evalill Bølstad [as part of previous studies: Bølstad et al. (2021, 2025) and then adapted for delivery with teachers (TIKiS: Tuning in to Kids in Schools) by Evalill Bølstad and Frederik Ferstad Skoe]. As each of the six teacher sessions were limited to only 90 min, compared to 120 min in the parenting program, some exercises were cut, and some shortened. In addition, there were adaptations made to reflect both the differences in relationship between a teacher-student- and parent- child, as well as the contextual differences in emotion coaching in a school versus family context. Some optional exercises that were more appropriate for parents were also removed. The most concrete example of this was the creation of scripted role plays for teacher-child interactions. In addition, emotion coaching was also encouraged to only be used in one-to-one interactions rather than with students in front of their peers or the whole class. Additional materials were also added to support the school implementation of the intervention.

The TIKiS intervention consisted of a 3-h introductory seminar for the school leaders and all teachers from Grade 1–7, 6 × 90-min group sessions for teachers in Grades 1–4, and a further 3 × 90-min implementation support sessions for the school leaders. Details of each of these components is outlined below.

#### TIKiS introductory seminar

The initial 3-h introductory seminar for all school staff was held before the start of the school year. The seminar was given to all staff to ensure wide support and understanding of the TIKiS approach which is part of increasing consistency of responses throughout the school environment. The seminar was led by certified Tuning in to Kids facilitators, including the second author. Content included teaching about children’s emotional competence (emotion expression, understanding, empathy and emotion regulation), the role of temperament, the relationship between needs and emotions, beliefs about emotions (meta-emotion), the different ways adults respond to children’s emotions (e.g., emotion dismissing, coaching styles), the role of self-care and the emotions that occur in a school setting.

#### TIKiS group sessions

In the end of September and beginning of October 2023 and 2024, group delivery of TIKiS began with teachers in Grades 1–4. The group sessions were delivered by trained facilitators using a structured TIKiS manual. Each session included handout materials and followed a structured outline that included introductory exercises, group reflection, roleplays, teaching of concepts, exercises and home activities. Teachers attended six group sessions, delivered once every 2 weeks for 90 min during the Autumn semester in the first half of the school year (September to December). The groups consisted of 6–8 teachers in Year 1 and required a minimum of four participants to be delivered. Due to significant illness and absenteeism, group sizes were increased to 8–12 teachers in Year 2. Teachers who were not present for sessions were sent materials from the session and asked to read this prior to the next session. They could also receive a summary of the session over the phone, in person or via email from the TIKIS facilitator.

TIKiS was delivered in the teachers’ workplace during their working hours. School leaders scheduled the dates, times and rooms for intervention delivery that were suitable for the teachers. The leaders were responsible for ensuring that the teachers knew when and where to meet for each session, and a register of attendance was kept by the TIKiS facilitators.

TIKiS used roleplays in each session to draw on teacher’s experiences with students and practice how emotion coaching could be used during these interactions. Roleplays included use of scripted roleplays that contrasted emotion dismissing with emotion coaching response styles, fishbowl roleplays where the whole groups engaged in the roleplay; and pairs/threes doing unscripted roleplays using teacher’s own examples to practice the skills with support from facilitators. Additionally, teachers were encouraged to use the ideas taught in the sessions in their interactions with students during the week and were given home activities between each session to build skills. See Table 2 for details on session content.

**Table 2:** TIKiS program content.

Session	Session content
Session 1: development of emotional competence	Introduce group members to each other, and to concepts of emotional competence and Emotion Coaching. Group guidelines and focus on group cohesion and engagement.
Session 2: tuning in to emotions and meta-emotion philosophy	Focus on naming emotions and noticing emotions at low levels, in individual students, and awareness of potentially triggering situations. Introduce concepts of meta-emotion philosophy.
Session 3: understanding students’ emotional experience	Focus on empathy and the importance of an emotion-focused and empathic response before problem solving.
Session 4: emotion coaching, self-care and responding to fear and worries	Focus on emotion coaching fear, anxiety and worry. Teacher self-care and wellbeing strategies.
Session 5: emotion coaching and strong expressions of emotion	Teacher emotion regulation. Focus on emotion coaching anger. Understand when emotion coaching is not appropriate.
Session 6: emotional competence now and ahead	Summarize main concepts learned including emotional competence, emotion coaching, and meta-emotion philosophy. Delivery of any exercises not covered. Practice core skills. Conclusion.

#### TIKiS implementation support sessions

School leaders received three 90-min group sessions to support teachers learn and apply emotion coaching skills. The sessions were led by certified TIKiS facilitators, who were part of the project team and experienced employees in pedagogical psychological services and the school health services, respectively. This was to ensure school leader engagement and long-term sustainability of the intervention, and to help the school leaders support their teachers to implement the TIKiS content.

### TIKiS group delivery—facilitators, training and supervision

#### TIKiS facilitators

TIKiS group facilitators were recruited from the municipal school health services (nurses in the school health services, *Skolehelsetjenesten*), city-wide educational-psychological services (pedagogues and psychologists from *Pedagogisk psykologisk tjeneste*) and municipal mental health services (*Oslohjelpa*), to deliver the 6-session TIKiS groups to teachers. Each TIKiS group was delivered by two facilitators, one from school health services and one from pedagogical psychological services. These employees have complementary competences and roles in schools. The municipal services had a more mental-health-oriented perspective, and mostly had familiarity with the schools they were working in. The facilitators from the educational-psychological services had expertise with group delivery with teachers, as well as understanding the teacher’s role in the classroom. Additionally, they often work with the same students without collaborating with each other, therefore the project aimed to improve the relationship between the two institutions as a wider goal for the intervention. All TIKiS facilitators had at least a master’s degree in psychology, pedagogy, special pedagogics or similar.

#### TIKiS facilitator training and supervision

All TIKiS group facilitators completed a two-day TIKiS training over 16 h. Facilitators received training in March 2023 and in May 2024, a few months before intervention delivery. This training was similar to the Tuning in to Kids facilitator training but with adaptations for running groups with teachers in the primary school setting. Training included the program philosophy, research and the content for each of the six group sessions plus how to deliver all the main exercises and processes of the program.

Following training, an additional collaboration workshop was held shortly before the TIKiS groups began with the second author (Skoe). This 90-min workshop explored how to enhance the relationship between the school health services and the pedagogical psychological service employees as they worked together to deliver the intervention to the groups, how to ensure fidelity to the program manual, working with teacher’s motivation and engagement, and mastery of the group facilitator role. The workshop summarized the core elements of TIKiS and included discussion and roleplay practice to ensure facilitators were competent with using roleplay.

Supervision was provided for facilitators by the second author on three occasions during the delivery of each TIKiS group: once immediately prior to session 1; after session 2 and after session 4. Supervision addressed the content being delivered in the sessions, explored issues and challenges arising (i.e., resistance to the program content), and built the understanding of the facilitators about core program elements (such as the role of meta-emotion philosophy, how to support teacher’s self-care or building the emotion coaching skills).

## Measures

### Effectiveness measures

Teachers completed baseline questionnaires in the week before school started in August, and at follow-up, approximately 10 months post-baseline measurement, in April/May of the following year. CLASS observations of teachers were conducted at similar time points.

#### Emotion socialization

This study used a modified version of the Coping with Children’s Negative Emotions Scale (CCNES; Fabes et al., 1990) to measure emotion socialization practices by exploring teacher’s reactions to student’s negative emotions. The questionnaire was originally designed for parents and translated to Norwegian by psychologists for a study of TIK with parents of kindergarten aged children (Bølstad et al., 2021). This modified measure was adapted for use with Norwegian teachers (CCNES-Teacher: Bølstad et al., 2023) and shortened to 35 items from the original 72 because teachers were not compliant with long questionnaires in the pilot study. Additionally, some of the subscales were changed or removed to be consistent with other TIK studies to improve validity of the sub-scales, and to ensure the CCNES was more culturally appropriate. Two subscales were added: Empathy/Acknowledgement of emotions, and Distraction.

Empathy/Acknowledgement is consistent with Gottman et al. (1996) concepts of the 5 steps of emotion coaching, and Distraction, measures teachers’ use of distraction as a strategy for responding to children’s emotions—also found by Gottman et al. (1996) to be an emotionally dismissive response style. In addition, the Punitive Reactions, Distress Reactions and Emotion Focused Subscales were removed because of poor validity (Bjørk et al., 2020; Bølstad et al., 2021). Subscales were combined into two dimensions measuring supportive or non-supportive emotion socialization practices. The Emotion Coaching (supportive) dimension consisted of Empathy/Acknowledgement, Expressive Encouragement and Problem Solving; the Emotion Dismissing dimension consisted of Minimization Reactions and Distraction. Using confirmatory factor analysis, the 35-item version of the CCNES was found to have acceptable reliability and validity. For more details see Koleini et al. (2026).

In the 35-item version, CCNES presents seven scenarios, with five different responses to each scenario, so each response corresponds to one of the five emotion socialization subscales. Teachers were asked to respond with the likelihood they would respond with each reaction on a scale of 1 (*very unlikely*) to 7 (*very likely*). For example, “If a student’s jacket has become damaged during recess, and they have become angry and disruptive, I would (a) help the student find out how the jacket can be repaired (Problem Solving), (b) support the student in the fact that it is terrible to have your jacket damaged (Empathy), (c) ask the student to calm down and say that the jacket can be repaired (Minimization Reactions), (d) encourage the student to express their anger in words (Expressive Encouragement) or (e) ask the student if they have any fun plans for the weekend (Distraction).

#### Teacher affect integration

The Affect Integration Inventory 18 (AII; Solbakken and Monsen, 2017) was used to measure teacher’s affect integration, which is the ability to understand and incorporate emotional experiences into well-adjusted cognition and behavior. The measure captures emotion awareness and regulation of the adult, but rather than dampening or restricting emotions it involves the capacity to allow oneself to experience the emotion and express it appropriately. Higher levels of affect integration can protect against psychopathology (Monsen et al., 1996) and has been used as a measure of psychological function and predictor of responsiveness to interventions. The AII presents 18 statements and asks the participant to rate how well these statements describe them from 0 (*does not fit at all*) to 9 (*fits completely*). For example, “When something very sad happens, I can cry and feel very relieved afterwards.” Psychologist Bølstad shortened the AII 3.1 from 112 questions to 18 questions for this study using factor analyses conducted by one of the authors of AII (Solbakken). The AII was shortened to 18 items to prevent questionnaire fatigue.

The AII questions load to multiple constructs, and the shortened version consists of three higher order scales, global affect integration (18 items), capacity for affect experience (11 items), and capacity for affect expression (7 items), as well as six discrete affect scales with three items each; integration of joy, integration of anger, integration of tenderness, integration of sadness, integration of shame and integration of jealousy. The AII is a Norwegian scale and did not require translation. Good internal reliability, structural validity, construct validity and discriminant validity for the scale has been established for the 42-item version of AII in Norway (Solbakken and Monsen, 2021). The AII-18 factors had acceptable internal reliability and factor validity (Koleini et al., 2026).

#### Teacher satisfaction, burnout and distress

This study used three scales to measure teacher life satisfaction, burnout and distress.

The Satisfaction with Life Scale (SWLS; Diener et al., 1985) is a 5 item questionnaire that measures life satisfaction. The measure presents five statements and participants rate how much they agree with each statement on a scale of 1 (*does not fit*) to 7 (*fits perfectly*) providing a total score. An example item includes, “I am content with my life”. SWLS shows good fit, internal reliability and convergent validity in various samples (Chinni and Hubley, 2014; Moksnes et al., 2014).

The Bergen Burnout Inventory (BII; Matthiesen, 1992; Salmela-Aro et al., 2011) is a 9-item measure of burnout, with three subscales, Exhaustion, Cynicism and Inadequacy, with three items in each. On a scale of 1 (*completely disagree*) to 6 (*completely agree*), participants were asked how much they agree with the following nine statements. For example, “I feel overwhelmed at work”. The English version of BBI was obtained from Salmela-Aro et al. (2011), translated to Norwegian and back translated to English by master’s students in psychology. BBI had showed good fit, internal reliability, and predictive validity for Estonian and Finnish managers (Salmela-Aro et al., 2011).

The Hopkins Symptoms Checklist 10 (HSCL-10; Derogatis et al., 1974; Hesbacher et al., 1980; Strand et al., 2003) is a 10-item measure of distress. On a scale of 1 (*not at all*) to 4 (*a lot*), participants are asked how much they have experienced the following symptoms in the past week. For example, “Feeling anxious”. HSCL-10 is based on HSCL-25 which was translated from English to Norwegian by Hesbacher et al. (1980) and later shortened to 10 items by Strand et al. (2003). HSCL-10 consists of two subscales, depression, and anxiety, with 6 and 4 items, respectively, that together create distress. HSCL-10 has shown good internal reliability, structural validity and external validity among Scandinavian adolescents (Haavet et al., 2011; Kleppang and Hagquist, 2016).

#### Classroom emotional climate

The Classroom Assessment Scoring System K3 (CLASS; Pianta et al., 2008) was used as an independent observation measure of the classroom emotional climate. CLASS consists of three domains, emotional support, classroom organization and instructional support, with three or four dimensions in each. Emotional support is the domain that most closely captures emotion climate and reflects the emotional connection and social interactions between teachers and students, the teacher’s awareness, and ability to effectively address their students’ emotional and academic needs, and the teacher’s interest in the student’s opinions. Teachers were scored on a scale of 1 (*low range, few if any expected behaviors are seen*) to 7 (*high range, many expected behaviors are seen most of the time*) for each dimension depending on the frequency and quantity of behaviors seen.

Three modifications were made to CLASS for the current study. First, teachers were observed in four cycles instead of six. This was due to a limited number of observers Second, teachers did not receive feedback after being observed because CLASS was used as an assessment tool and not as a method of intervention, which it is often used as, as this would make it harder to discern whether changes in behavior were due to TIKiS, or due ot the feedback. Last, if there was more than one teacher in the classroom, observers were instructed to focus on the lead teacher who was receiving the TIKiS intervention. This was to ensure that changes observed in the classroom were due to changes in the teacher who received TIKiS, and not because of other teachers.

Observers were a mix of employees from pedagogical psychological services, master and bachelor students in psychology, pedagogic psychological counseling, and special pedagogics. They all participated in 14 h of certified training by Teachstone and had to pass an exam with 80% reliability. Additionally, Interrater reliability was computed for 10% of all CLASS observations with double coding by a second coder. All coders were blind to the condition of the class (whether they were intervention or control).

### Intervention implementation

Implementation of TIKiS was maximized through use of structured manuals, implementation support session with school leaders, supervision, and additional support for the group facilitators throughout the intervention. This ensured high quality fidelity of intervention delivery. Implementation was measured on five of eight dimensions suggested by Durlak and DuPre (2008) and Humphrey et al. (2016). The dimensions were program differentiation, reach, fidelity, dosage, and adjustment including teacher feedback.

#### Program differentiation

Program differentiation indicated how different TIKiS was from other interventions or programs that the teachers had previously or were currently exposed to Durlak and DuPre (2008) and Humphrey et al. (2016). Program differentiation helps determine whether observed effects are masked by involvement in similar previous interventions. Program differentiation was measured using a scale developed for this study, drawing inspiration from questionnaires used for other interventions, such as the ROBUST school program (Rege et al., 2025). At baseline, participants were asked on a scale of 1 to 5, to what extent they had worked systematically with strategies or measures that focused on “verbal and nonverbal communication”, “student behavior”, “relaxation techniques”, “emotions”, “social competence”, “relationships between teachers and students”, “interacting with children when they are emotional”, or “teachers’ own emotional wellbeing” during the previous year. The measure consisted of 8 items, one for each strategy. A summed total score for program differentiation was calculated.

#### Reach

Reach is how many people the intervention reached, and how representative the sample was of the rest of the population. This was measured by the proportion of eligible schools and teachers that participated in the intervention, and how different they were from those who did not participate. as well as the proportion of eligible teachers in a school who participated in the intervention. Except for schools that had participated in the pilot study, all public elementary schools in Oslo were invited to participate in the intervention.

#### Fidelity/adherence

Implementation fidelity measures whether the intervention was delivered as intended. This study examined content fidelity or intervention adherence, which measures how much of the predetermined content was covered. After each TIKiS group session, facilitators completed a checklist indicating whether core components had been covered.

#### Dosage and attendance

The dosage is a measure of how much of the intervention school leaders and teachers received. Dosage was measured by facilitators taking attendance for each session with school leaders and teacher groups, and by calculating the percentage of sessions teachers attended. Attendance at the introductory seminar was recorded separately in the baseline questionnaire. Teachers and school leaders who were present at the 3-h introduction seminar completed the baseline questionnaire prior to the seminar, providing a tally of attendance.

#### Adjustment

Adjustment or adaptation refers to changes that were made to the intervention during the implementation period. Deviations in content adherence were collected using a checklist questionnaire filled in by the facilitators after each session, and other differences from the manual were noted down qualitatively as they occurred.

#### Acceptability, feasibility, and appropriateness

Acceptability, feasibility and appropriateness were measured using self-report validated questionnaires, intervention feedback questionnaires, and focus group interviews.

The self-report questionnaire used the Acceptability of Intervention Measure (AIM), the Intervention Appropriateness Measure (IAM) and the Feasibility of Intervention Measure (FIM), which are three questionnaires used to measure acceptability, feasibility, and appropriateness (Weiner et al., 2017). Each measure presents five statements, and participants indicate how much they agree with each statement on a scale of 1 (completely disagree) to 5 (completely agree). For example, “TIKiS meets my approval”. The AIM, IAM and FIM questionnaires were administered post intervention in April.

After each TIKiS group session both teachers and facilitators answered a brief feedback questionnaire where they are presented with five statements asking to what degree they agreed to each statement on a scale of 1 (not at all) to 5 (to a large extent). The questionnaire evaluated whether the session material was useful/not-useful, easy/difficult, helped teachers respond to students’ emotions, helped teachers be better at handling their own emotional reactions at school, and whether teacher’s believed emotional competence was important at school.

Qualitative interviews were conducted after the intervention with school leaders to provide greater depth in understanding of school staff experience of the intervention.

#### Focus group interviews

In addition to the quantitative measures, participants were recruited to take part in focus group interviews to explore their experience of the intervention, leadership support, collaborative relationships and perceived school leader ownership of the implementation of TIKiS. The intent of the focus group interviews was to have a more exploratory, bottom-up way to uncover themes and experiences regarding the implementation process that could supplement the existing quantitative measures. There were two focus group interviews: one for the facilitators, and one for those in the school leadership. For the leadership interviews, recruitment was conducted via email with the school contact and principal. Recruitment for the facilitator interviews was done by contacting the facilitators directly. Interviews were conducted online, lasted up to 90 min and were conducted by a clinical psychologist with whom the participants had no prior contact.

## Data management

Data was stored in Tjenester for Sensitive Data (TSD; *Services for Sensitive data*), a platform designed, maintained and owned by the University of Oslo IT-Department for storing and post-processing of sensitive data. TSD provides virtual servers and a large set of software tools confined within a highly secured environment. Additionally, a PGP encrypted version of the UIO web-questionnaire allows data to be securely stored directly to a TSD project area. This way data is fully contained within TSD, and only removed once it has been fully anonymised or aggregated. Access to TSD required a national ID to log in with the governmental ID-portal, and the approval of the TSD project manager. Only the TSD project manager could export data out of TSD. To ensure the anonymity of participants, only aggregated data was published.

## Analyses

Analyses were done in R (R Core Team, 2021) using linear mixed models with the package lme4 (Bates et al., 2015), where we looked at the interaction between condition (i.e., waitlist control or intervention dummy coded as 0 and 1) and ‘time’ (i.e., baseline and follow-up dummy coded 0 and 1), and with teacher as random intercept.

Missing data will be handled using multiple imputation by chained equation using the mice package (van Buuren and Groothuis-Oudshoorn, 2011), and full maximum likelihood in the lmer function.

Because teachers are nested within schools, intraclass correlations (ICC) will be calculated for each of the baseline variables. The intraclass correlation provides a sense of the degree to which differences in the outcome variable exist between Level 2 units (i.e., schools). If ICC exceeds 0.05, multi-level analyses are warranted to control for the impact of the nesting.

Using Calculator for Two-Level Cluster Random Assignment Design (CRA2_2r) from PowerUP (Version: 05/12/2019; Dong and Maynard, 2013) with *α* of 0.05, power of 0.80 and ICC of 0.05, it was calculated that we have the power to find effect sizes of 0.39 if we have 20 schools with 20 teachers each (400 teachers total).

## Discussion

This protocol outlined the Tuning in to Kids in School (TIKiS) study conducted in elementary schools in Oslo during 2023 to 2025, and the measures that were used to assess the implementation and effectiveness of the intervention. TIKiS aims to improve emotional competence in children by improving the emotion socialization practices of teachers. Supporting teachers to respond effectively to emotions in the classroom context is an important opportunity for promoting children’s emotional competence. These skills are associated with better developmental outcomes in children, such as higher teacher-rated learning outcomes (Martinsone et al., 2022) and lowered risk of negative life outcomes such as poor mental health (Blewitt et al., 2018; Greenberg et al., 2017) and externalizing behavior (Espejo-Siles et al., 2020; You et al., 2023). These skills are also important for teacher’s own emotional wellbeing (Aldrup et al., 2020; Oliveira et al., 2021). Teachers are important emotion socializers who can help improve the emotional competence of children through emotion coaching (Morris et al., 2013) in a universal intervention.

Some of the strengths of this study are that it is a randomized controlled trial recruiting 20 schools and 200 + teachers. The design of this study was based on a pilot trial of TIKiS with primary school teachers that allowed refinement of methods and intervention. The study used multiple methods to test effectiveness and implementation, such as self-report questionnaires, observations, and qualitative interviews. The study used condition-blind observers who had been trained in standardized CLASS measurement improving the objectivity of the study evaluation.

The design of this study also had some limitations. The teachers attended the whole school TIKiS introductory seminar before the school year started, and before baseline CLASS observations had occurred. Therefore, the teachers were already exposed to a small dose of the intervention before the first observations were conducted. This introduction may have already affected the way teachers were responding; however, this approach was used because it would have been too difficult for the teachers to attend the introductory seminar once the school year had started, and children were in attendance (when it was possible to observe the teachers interacting with the children using CLASS). There was only 4–5 months between the intervention finishing and follow-up data collection; a longer follow-up would have been ideal to see whether the changes were sustained; however, then there would be a new school year with different teachers and children in classrooms. Measures of children’s functioning would also have been ideal but were beyond the study design; seeking consent from every parent for child assessment would have been very difficult resulting in a bias in who data was gathered from. A final limitation was that data were not collected on how or why schools chose to include special education teachers in the study. This was due to differences in whether schools considered special education teachers to be ‘regular teachers’, or due to differences in what services these schools provided. This made it harder to evaluated the benefits of TIKiS on special education teachers, and may have resulted in some special education teachers being excluded from participation by the school.
